# Relationship of Hepatocellular Carcinoma Stage and Hepatic Function to Health-Related Quality of Life: A Single Center Analysis

**DOI:** 10.3390/healthcare11182571

**Published:** 2023-09-18

**Authors:** Amol Gupta, Jane Zorzi, Won Jin Ho, Marina Baretti, Nilofer Saba Azad, Paige Griffith, Doan Dao, Amy Kim, Benjamin Philosophe, Christos Georgiades, Ihab Kamel, Richard Burkhart, Robert Liddell, Kelvin Hong, Christopher Shubert, Kelly Lafaro, Jeffrey Meyer, Robert Anders, William Burns III, Mark Yarchoan

**Affiliations:** The Sidney Kimmel Comprehensive Cancer Center, The Johns Hopkins Hospital, Baltimore, MD 21287, USA; jzorzi1@jhmi.edu (J.Z.); wjho@jhmi.edu (W.J.H.); mbarett1@jhmi.edu (M.B.); nazad2@jhmi.edu (N.S.A.); pgriff18@jhmi.edu (P.G.); ddoa1@jhmi.edu (D.D.); akim97@jhmi.edu (A.K.); bphilosophe@jhmi.edu (B.P.); cgeorgi@jhmi.edu (C.G.); ikamel@jhmi.edu (I.K.); burkhart@jhmi.edu (R.B.); rliddel1@jhmi.edu (R.L.); khong1@jhmi.edu (K.H.); christopher.shubert@jhu.edu (C.S.); klafaro1@jhmi.edu (K.L.); jmeyer58@jhmi.edu (J.M.); rander54@jhmi.edu (R.A.); wburnsi1@jhmi.edu (W.B.III); mark.yarchoan@jhmi.edu (M.Y.)

**Keywords:** hepatocellular carcinoma, health-related quality of life, Child–Pugh score

## Abstract

Health-related quality of life (HRQoL) is known to be an important prognostic indicator and clinical endpoint for patients with hepatocellular carcinoma (HCC). However, the correlation of the Barcelona Clinic Liver Cancer (BCLC) stage with HRQoL in HCC has not been previously studied. We examined the relationship between BCLC stage, Child–Pugh (CP) score, and Eastern Cooperative Oncology Group (ECOG) performance status on HRQoL for patients who presented at a multidisciplinary liver cancer clinic. HRQoL was assessed using the Functional Assessment of Cancer Therapy-Hepatobiliary (FACT-Hep) questionnaire. Fifty-one patients met our inclusion criteria. The FACT-Hep total and subscales showed no significant association with BCLC stages (*p* = 0.224). Patients with CP B had significantly more impairment in FACT-Hep than patients with CP A. These data indicate that in patients with HCC, impaired liver function is associated with reduced quality of life, whereas the BCLC stage poorly correlates with quality of life metrics. Impairment of quality of life is common in HCC patients and further studies are warranted to determine the impact of early supportive interventions on HRQoL and survival outcomes.

## 1. Introduction

Primary liver cancer is the sixth leading cancer type and the fourth leading cause of cancer-related mortality worldwide. In 2020, 906,000 new cases were reported, with 830,000 deaths [1]. Hepatocellular carcinoma (HCC) accounts for approximately 85% of cases of primary liver cancer and is projected to be the third leading cause of cancer-related mortality by 2030 [2]. The HCC treatment landscape has changed rapidly over the last decade due to the introduction of many novel therapies, including systemic immunotherapies [3]. However, the outcome of HCC still lags behind many other malignancies, which may be partly due to the advanced stage at presentation and the associated comorbidity of liver cirrhosis [4]. Patients who commence current systemic therapy regimens for advanced-stage HCC have at best a median overall survival (OS) of approximately 19 months [5,6].

Several factors drive the prognosis of HCC, including liver function and performance status. The Barcelona Clinic Liver Cancer (BCLC) staging system is the most widely used staging system for HCC. It combines tumor stage, liver function, and performance status to stratify patients into five stages [7]. Current clinical guidelines consider BCLC the most useful staging system for assessing patient status and treatment selection [8]. The Child–Pugh scoring system is an essential component of BCLC staging, which classifies patients into three grades of hepatic impairment [9]. Additionally, the Eastern Cooperative Oncology Group (ECOG) performance status is a strong predictor of survival in HCC patients [10].

There has recently been increasing interest in health-related quality of life (HRQoL) as a vital indicator of treatment efficacy and tolerability in cancer patients. Current evidence suggests that the HRQoL may be important as survival and should be regarded as a clinical endpoint [11,12]. The information provided by HRQoL assessment at diagnosis can optimize the risk–benefit evaluation and help in guiding treatment selection according to patient needs and preferences.

Especially in patients with HCC, the HRQoL can be affected by underlying liver function, treatment-related complications, associated psychological distress, and ability to undertake daily activities [13]. Impairment of HRQoL has been demonstrated in patients after hepatic resection due to the post-operative morbidities and the high tumor recurrence rate. Also, post-chemoembolization pain can impair HRQoL in patients with HCC [12]. However, HRQoL can be affected as early as disease diagnosis due to the symptomatic burden of liver cirrhosis with associated findings of fatigue, abdominal pain, anorexia, ascites, gynecomastia, pruritis, sexual dysfunction, and related comorbidities [12,14,15]. These symptomatic burdens can also lead to distress and several psychological disorders, further impairing the HRQoL of the patients [16]. The HRQoL role in the HCC patient does not only involve treatment selection and patient well-being but also patient prognosis and survival. Previous reports showed that HRQoL has a prognostic value in HCC and other malignancies, with a significant association between impaired HRQoL and worse survival outcomes [17,18,19].

Several studies have assessed the clinical use and application of the self-reported Functional Assessment of Cancer Therapy-Hep (FACT-Hep) questionnaire in HCC patients; however, the correlation of BCLC staging with HRQoL in HCC has not been studied. We utilized real-world data from a single institution to investigate the relationship between the HRQoL at diagnosis and BCLC stage, Child–Pugh score, and the ECOG performance status of 51 patients with HCC using the FACT-Hep questionnaire. We hypothesized a significant correlation between BCLC stage, Child–Pugh score, and ECOG performance status and HRQoL, and to understand whether any of these disease measures could serve as a proxy for HRQoL in treatment decision-making.

## 2. Materials and Methods

### 2.1. Study Design and Eligibility Criteria

Patients presenting with HCC at the Johns Hopkins Hospital Liver Multidisciplinary Clinic are routinely asked to complete the self-reported FACT-Hep questionnaire to assist healthcare providers in identifying and addressing quality-of-life issues. These surveys were usually administered at the start of the visit, before discussions with the treatment team about the diagnosis and treatment plan. Patients who were severely encephalopathic, and thus potentially unable to understand written English, were not administered the survey in routine practice. A retrospective medical chart review was undertaken of patients with HCC who presented to the multidisciplinary liver clinic at the Johns Hopkins Hospital between February 2020 and August 2022, and had previously completed a FACT-Hep questionnaire during their visit. Patients with missing or incomplete FACT-Hep survey scores, or incomplete staging or liver function status were excluded.

### 2.2. Data Collection

The following data were retrieved from the records of eligible patients: demographic characteristics; diagnosis of HCC; method of diagnosis; BCLC stage; ECOG performance status; Child–Pugh class; causes of liver disease; and FACT-Hep scores.

The Child–Pugh score comprises five criteria: albumin, bilirubin, INR/PTT, hepatic encephalopathy, and ascites. The Child–Pugh score was calculated using the most recent laboratory testing available before or on the day of the patient’s clinical evaluation.

The generic Functional Assessment of Cancer Therapy (FACT-G) is a 27-item tool that evaluates cancer patients’ functional, physical, social, and emotional well-being [19]. In 2002, FACT-Hep was developed to measure the extent of the HRQoL impairment in patients with hepatobiliary cancers [20,21]. The FACT-Hep self-reported disease-specific questionnaire aims to assess general and disease-specific aspects of patient HRQoL (20). The FACT-Hep survey assesses five domains that include physical well-being (PWB), such as pain; social well-being (SWB), including social, emotional, and sexual well-being; emotional well-being (EWB); functional well-being (FWB) that focuses on work and activities of daily living; and the hepatobiliary cancer subscale (HCS) that includes hepatobiliary symptoms. Each domain is composed of a five-point Likert scale from 0 to 4. The FACT-Hep total score (range, 0–180) was calculated by combining the sub-scores of the five domains. The first four domains were used to calculate the FACT-G score (range, 0–108), while the PWB, FWB, and HCS were used to calculate the FACT-Hep Trial Outcome Index (range, 0–128) [22].

### 2.3. Statistical Analysis

Descriptive analysis was employed according to the data type and normal distribution. One-way analysis of variance (ANOVA) and independent *t*-tests were used to compare the association between the FACT-Hep score or sub-scores and the BCLC staging system, Child–Pugh score, and ECOG performance status. Data were analyzed using JMP version 15.2 (SAS Inc., Cary, NC, USA), with a two-sided significance of 0.05.

## 3. Results

### 3.1. Demographic Data, Staging/Classification, and Fact-Hep Scores for the Overall Cohort

A total of 56 patients were given the survey; of them, five surveys were partially filled out and so the results were excluded. Fifty-one patients with HCC met inclusion criteria with a median age of 69 years (range 62.5–75.0 years). Most patients were male (85.7%). The most common cause of cirrhosis was hepatitis C virus (HCV) infection, followed by nonalcoholic steatohepatitis/nonalcoholic fatty liver disease (NASH/NAFLD). Most patients (80%) had received no prior treatment. At the time of presentation, 41.2% of the patients had BCLC stage A, 25.5% had BCLC stage B, and 33.3% had BCLC stage C. No patients had BCLC stage D disease in this retrospective study cohort. The majority of the patients had Child–Pugh class A (75%) and ECOG 0 (54.9%). The mean FACT-Hep total score for the overall cohort was 130.3 ± 20.3; 23 patients (45.1%) had a total FACT-Hep score of less than 130 (Table 1).

### 3.2. Association between FACT-Hep Scores and BCLC, ECOG, and Child–Pugh at Diagnosis

**Figure 1:**
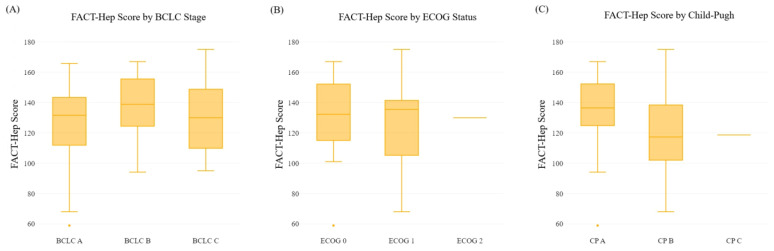
Boxplots of the association between the FACT-Hep total score and (**A**) the BCLC stage categories, (**B**) the ECOG performance status, and (**C**) the CP class. Abbreviations: FACT-Hep, Functional Assessment of Cancer Therapy-Hepatobiliary; BCLC, Barcelona Clinic Liver Cancer; CP, Child–Pugh; ECOG, Eastern Cooperative Oncology Group.

We examined the relationship between the HCC stage and HRQoL scores. Overall FACT-Hep scores were comparable for patients with BCLC stage A, B, and C disease (*p* = 0.224). However, advanced stage HCC (BCLC C) was associated with significantly increased impairment in the EWB subdomain scores compared with patients with BCLC stage B and A (18.2 ± 3.4 versus 16.1 ± 4.5 and 14.5 ± 4.9, respectively; *p* = 0.001). Other FACT-Hep sub-scores were similar across the BCLC stages (Table 2, Figure 1).

We next evaluated the relationship between ECOG performance status and HRQoL scores. The analysis showed no impact of ECOG performance status on the FACT-Hep total score or sub-scores, except for the FWB sub-score. Patients with an ECOG performance status 1 had significantly more impairment in the FWB than patients with status 0 (14.5 ± 6.5 versus 18.1 ± 6.2, respectively; *p* = 0.006) (Table 2, Figure 1).

When we assessed the association between Child–Pugh class and FACT-Hep domains, we found that patients with Child–Pugh class B had significantly more impairment in FACT-Hep domains than patients with Child–Pugh class A, including the FACT-Hep total score (118.5 ± 27.3 versus 134.3 ± 23.8; *p* = 0.021) and sub-scores [PWB (*p* = 0.018), FWB (*p* = 0.030), HCS (*p* < 0.001), FACT-Hep Trial Outcome Index score (*p* = 0.003)] (Figure 1). There was only one patient with Child–Pugh class C, with a FACT-Hep score of 118.6. Therefore, a statistical test comparison of Child–Pugh class C with other classes was not feasible. There were no significant associations between FACT-Hep total or sub-scores and age or gender (*p* > 0.05; Table 2, Figure 1).

## 4. Discussion

### 4.1. Key Findings

The present real-world study investigated the relationship between HRQoL at diagnosis and the BCLC stage, Child–Pugh score, and ECOG performance status of 51 patients with HCC using the FACT-Hep questionnaire. Our results demonstrated that in HCC, the BCLC stage poorly correlates with HRQoL. On the other hand, the severity of liver dysfunction (as measured via the Child–Pugh score) correlates well with the worse HRQoL of HCC patients. Our results also showed that the EWB was impaired in advanced-stage HCC, independent of functional or physical status.

### 4.2. Explanation and Comparison with Similar Research

Quality of life generally correlates to the stage of cancer [23]. Previous reports have demonstrated a significant association between advanced cancer stage and impairment in the HRQoL of patients with numerous malignancies, including esophageal cancer [24,25], breast cancer [26], and colorectal cancer [27]. The symptomatic burden and the limited availability of treatment options may explain the negative impact of advanced cancer stages on HRQoL. Additional factors, such as malnutrition and socioeconomic status, were found to impact the HRQoL of patients with advanced cancer [27,28]. The BCLC staging system is used to stage HCC patients and select treatment options [29]. It includes not only the Child–Pugh score but also tumor extent and ECOG performance status. To our knowledge, no previous studies have investigated the association between BCLC staging and the HRQoL of HCC patients. Our study observed that the BCLC stage was not significantly correlated with HRQoL. Such findings reflect that the BCLC staging system may be less sensitive to quality-of-life information. In BCLC stages A, B, and C, both Child–Pugh A and B are grouped together. However, we have seen that the quality of life between Child–Pugh A and B is significantly different. Thus, we may be underestimating the quality of life of patients when using BCLC staging.

We also found that patients with a more advanced-stage HCC had more impairment in their EWB, independent of their functional, physical, or social well-being. The symptoms of liver function impairments, including general fatigue, indigestion, persistent nausea, anorexia, and ascites, can exert a substantial psychological burden on patients and their families [30]. Notably, even before disease progression and the development of severe symptoms, the disclosure of an HCC diagnosis can disturb the EWB of patients and lead them to feel threatened, uncertain, fear death, and become psychologically unstable [31,32].

The Child–Pugh score is a commonly used indicator of liver dysfunction in patients with cirrhosis and a reliable predictor of mortality in patients with HCC [33]. The Child–Pugh score has been used as part of the eligibility criteria by regulatory agencies for the approval of current systemic therapies. The findings from our study showed that HCC patients with Child–Pugh class B had significantly increased impairment in HRQoL, as measured via the physical, functional, HCS, and FACT-Hep total scores, than patients with Child–Pugh class A. Our findings are supported by studies that have also shown a significant association between impairment in several domains of the FACT-Hep score and a higher Child–Pugh class [19].

The present study utilized the FACT-Hep to assess HRQoL in HCC patients. However, it is worth noting that several HRQoL tools are available in the HCC setting, including EORTC QLQ-C30. Different tools are constructed based on certain theories and assumptions about what constitutes HRQOL and how it should be measured. The EORTC QLQ-C30, for instance, was developed with a focus on both symptom scale and social domain [34]. Thus, it is plausible that one tool may be more sensitive or offer a more comprehensive assessment of social well-being than another. Additionally, some HRQOL tools may exhibit higher responsiveness in certain populations, like the EORTC QLQ-C30 for chemotherapy patients [35]. Since the choice of an HRQOL instrument can substantially influence results, further studies on HCC patients should aim to assess the association between severity scores and different HRQoL tools to understand the impact of tool selection on the results.

### 4.3. Implications and Actions Needed

In our analysis, BCLC stage correlates poorly with HRQoL. This finding suggests that incorporating HRQoL tools can provide additional, valuable insight for the treatment team that may not be captured using the BCLC staging system. Recognition of the HRQoL may potentially identify patients who could benefit from supportive interventions, including palliative care services, social support, and coping skills depending on the specific domain that is found to be affected. In addition, HRQoL data may also be helpful in choosing between two similar treatments for the patients with the same stage of cancer, which may have varying impacts on quality of life. Prospective trials are needed to determine if ascertainment of HRQoL can lead to improved treatment outcomes for patients.

Impaired liver function is associated with reduced HRQoL in HCC patients. Further large-scale prospective studies should be conducted to determine the impact of early supportive interventions on HRQoL and survival outcomes in patients with HCC presenting with impaired liver function.

### 4.4. Limitations

This study had several limitations. This was a single-center study at a tertiary academic center. The sample size was relatively small and had only a single patient with Child–Pugh class C and no patients with BCLC D HCC, likely reflecting referral biases to a clinic that is focused on therapeutic cancer interventions. It is worth noting that the distribution of Child–Pugh stages within the BCLC categories may have influenced our findings. Specifically, the heterogeneity of liver function status, as reflected by the Child–Pugh classification within BCLC stages, could potentially affect the patients’ HRQoL and thereby the FACT-Hep score. Therefore, it is possible that this heterogeneity has contributed to the observed lack of a significant association between BCLC stage and FACT-Hep score. There was limited availability of data on potential confounders that may affect the HRQoL domains in HCC patients. Additionally, patient overall survival could not be evaluated due to the short duration of follow-up [36]. Thus, further prospective studies with larger sample sizes are needed to validate our findings.

## 5. Conclusions

Cancer stage has been shown to correlate with quality of life in various cancers. However, in our limited experience with HCC patients, the BCLC stage poorly correlates with HRQoL at diagnosis. We recommend incorporating a validated HRQoL tool like FACT-Hep alongside BCLC staging in the routine assessment of HCC patients. Impaired liver function is associated with reduced HRQoL. Further studies are warranted for HCC patients with liver dysfunction which assess the impact of supportive interventions on HRQoL and survival outcomes. Additionally, future research may benefit from incorporating stratification using the Child–Pugh stage within each BCLC stage to assess potential differences in HRQoL. However, our findings are limited by the retrospective nature of the study and the small sample size. Further prospective studies with larger sample sizes are needed to validate our findings and provide a better understanding of how liver cancer staging and hepatic function interact to influence patients’ quality of life.

## Figures and Tables

**Table 1:** Demographic data and the Barcelona Clinic Liver Cancer (BCLC) Stage, Child–Pugh Class, and the Eastern Cooperative Oncology Group (ECOG) performance status on the Health-Related Quality of Life (HRQoL) of 51 patients with hepatocellular carcinoma (HCC) using the Functional Assessment of Cancer Therapy-Hepatobiliary (FACT-Hep) questionnaire (n = 51).

**Age, y**	Median (IQR)	69 (62.5 to 75)
**Sex, n (%)**		
	Male	37 (72.5)
	Female	14 (27.5)
**Diagnostic method, n (%)**		
	Pathological	24 (47.1)
	Radiological	27 (52.9)
**Liver disease etiology, n (%)**		
	No liver disease	6 (11.8)
	Hepatitis C	15 (29.4)
	Hepatitis B	1 (2.0)
	NASH/NAFLD	15 (29.4)
	ETOH	5 (9.8)
	Other	4 (7.8)
	Unknown	5 (9.8)
**BCLC stage, n (%)**		
	A	21 (41.2)
	B	13 (25.5)
	C	17 (33.3)
	D	0 (0.0)
**ECOG Performance Status, n (%)**		
	0	28 (54.9)
	1	22 (43.1)
	2	1 (2.0)
**Child–Pugh, n (%)**		
	A	38 (74.5)
	B	12 (23.5)
	C	1 (2.0)
Prior treatment, n (%) *		**(n = 50)**
	Locoregional	11 (22.0)
	Surgery	4 (8.0)
	Systemic	3 (6.0)
	Transplant	1 (2.0)
	None	40 (80.0)
**PWB**	Mean ± SD	21.3 ± 5.9
**SWB**	Mean ± SD	22.1 ± 5.1
**EWB**	Mean ± SD	16.2 ± 4.6
**FWB**	Mean ± SD	16.4 ± 6.6
**HCS**	Mean ± SD	54.3 ± 11.7
**FACT-Hep score**	Mean ± SD	130.3 ± 20.3
**FACT-Hep Trial Outcome Index**	Mean ± SD	92.4 ± 20.3
**FACT-G Score**	Mean ± SD	75.9 ± 16.7

NASH/NAFD: nonalcoholic steatohepatitis/nonalcoholic fatty liver disease; HCC: hepatocellular carcinoma; ETOH: ethyl alcohol; BCLC: Barcelona Clinic Liver Cancer; ECOG: Eastern Cooperative Oncology Group; FACT-Hep: Functional Assessment of Cancer Therapy-Hepatobiliary; PWB: physical well-being; SWB: social/family well-being; EWB: emotional well-being; FWB: functional well-being; HCS: hepatobiliary cancer subscale. * Patient may have more than one treatment.

**Table 2:** Associations between the Barcelona Clinic Liver Cancer (BCLC) Stage at diagnosis, the Eastern Cooperative Oncology Group (ECOG) performance status, Child–Pugh class, age, and gender on the Health-Related Quality of Life (HRQoL) of 51 Patients with hepatocellular carcinoma (HCC) using the Functional Assessment of Cancer Therapy-Hepatobiliary (FACT-Hep) questionnaire.

Variables, Mean ± SD		PWB	SWB	EWB	FWB	HCS	FACT-G Score	FACT-Hep Trial	FACT-Hep Total
**BCLC stage**									
	A	21.7 ± 5.9	21.1 ± 5.4	14.5 ± 4.9	15.8 ± 6.1	52.8 ± 13.1	73.2 ± 18.1	91.4 ± 20.8	126.0 ± 28.1
	B	21.9 ± 6.3	23.7 ± 5.9	16.1 ± 4.5	16.7 ± 7.4	58.5 ± 8.4	78.4 ± 17.4	97.1 ± 18.7	136.9 ± 22.8
	C	20.4 ± 5.5	22.3 ± 3.7	18.2 ± 3.4	16.8 ± 6.5	52.8 ± 11.4	77.7 ± 13.8	90.0 ± 20.5	130.5 ± 22.7
	*p*-value ^a^	0.518	0.124	**0.001**	0.799	0.098	0.350	0.371	0.224
**ECOG Performance Status**									
	0	22.2 ± 5.3	21.5 ± 5.5	15.4 ± 4.5	18.1 ± 6.2	55.5 ± 10.4	77.2 ± 16.4	95.8 ± 19.3	132.6 ± 24.3
	1	20.1 ± 6.5	23.0 ± 4.7	17.2 ± 4.6	14.5 ± 6.5	52.5 ± 13.2	74.8 ± 17.2	88.0 ± 21.2	127.3 ± 27.0
	2	23.0	21.0	17.0	9.0	60.0	70.0	92.0	130.0
	*p*-value ^a^	0.174	0.364	0.140	**0.006**	0.354	0.680	0.166	0.577
**Child–Pugh**									
	A	22.2 ± 5.8	22.2 ± 4.7	15.8 ± 4.5	17.4 ± 6.6	56.7 ± 9.6	77.6 ± 16.7	96.3 ± 19.2	134.3 ± 23.8
	B	19.3 ± 5.3	21.7 ± 6.5	17.3 ± 4.8	13.5 ± 5.8	46.7 ± 14.6	71.8 ± 16.3	81.2 ± 19.8	118.5 ± 27.3
	C	14.0	25.6	15.0	13.0	51.0	67.6	78.0	118.6
	*p*-value ^a^	**0.018**	0.576	0.346	**0.030**	**<0.001**	0.255	**0.003**	**0.021**
**Gender**	Female	21.3 ± 5.7	22.3 ± 4.7	15.9 ± 5	15.7 ± 6.7	55.5 ± 12.9	75.2 ± 15.5	91.4 ± 22.3	129.6 ± 26.2
	Male	21.4 ± 6.0	22.1 ± 5.4	16.3 ± 4.5	16.6 ± 6.6	54.2 ± 11.4	76.3 ± 17.3	92.7 ± 19.8	130.5 ± 25.4
	*p*-value ^b^	0.961	0.916	0.838	0.677	0.944	0.824	0.856	0.918
**Age**	<65	20.3 ± 7.0	20.7 ± 6.3	16.6 ± 5.6	15.9 ± 7.3	55.1 ± 11.1	73.5 ± 21.3	91.3 ± 23.8	128.6 ± 30.8
	>65	21.7 ± 5.4	22.7 ± 4.6	16.0 ± 4.2	16.5 ± 6.4	53.9 ± 12.1	76.9 ± 14.8	92.7 ± 19.2	130.9 ± 23.5
	*p*-value ^b^	0.488	0.300	0.713	0.799	0.748	0.591	0.842	0.809

BCLC: Barcelona Clinic Liver Cancer; ECOG: Eastern Cooperative Oncology Group; FACT-Hep: Functional Assessment of Cancer Therapy-Hepatobiliary; PWB: physical well-being; SWB: social/family well-being; EWB: emotional well-being; FWB: functional well-being; HCS: hepatobiliary cancer subscale. ^a^ Using one-way ANOVA. ^b^ Using independent *t*-test.

## Data Availability

The datasets analyzed during the current study are available from the corresponding author on reasonable request.
